# No-Touch Saphenous Vein Graft for Coronary Artery Bypass Grafting

**DOI:** 10.5761/atcs.ra.25-00081

**Published:** 2025-08-16

**Authors:** Min-Seok Kim, Seong Wook Hwang, Ki-Bong Kim

**Affiliations:** 1 Cardiovascular Center, Myongji Hospital, Gyeonggi-do, Republic of Korea; 2 Hanyang University College of Medicine, Seoul, Republic of Korea

**Keywords:** coronary artery bypass grafting (CABG), no-touch saphenous vein (NT SV)

## Abstract

Compared with the conventionally harvested saphenous vein graft, the no-touch saphenous vein graft, in which manipulation and tension are minimized and intraluminal dilatation is avoided during vein harvest, has shown better-preserved luminal endothelium and improved patency rates after coronary artery bypass grafting. This review article will detail and summarize the relevant literature on the no-touch saphenous vein in coronary artery bypass grafting.

## Introduction

Since the introduction of the saphenous vein (SV) as an aortocoronary bypass graft in the early 1960s,^1,2)^ the SV conduit has been widely used for coronary artery bypass grafting (CABG). However, atherosclerotic failure of the SV conduit, lower long-term patency rates, and worse clinical outcomes have been reported after CABG performed with conventionally harvested SV aortocoronary grafts compared with CABG performed with internal thoracic artery (ITA) grafts.^3,4)^ Various methods and strategies, such as intraoperative pharmacologic treatment, external support devices, gene therapy, atraumatic “no-touch (NT)” SV harvesting techniques, the use of the SV as a composite graft based on the ITA, and postoperative optimal medical therapy, have been used to improve SV conduit patency.^5–12)^ The NT SV harvesting technique, in which manipulation during SV harvest is minimized and intraluminal dilatation is avoided, is one of those efforts. The 2018 European Guidelines for myocardial revascularization recommended that NT SV harvesting should be considered when an open technique is used (Class IIa, Level of Evidence B).^13)^ The purpose of this manuscript is to discuss recent surgical strategies and patency results of CABG using NT SV.

## Conventional SV Harvesting Techniques

Completely open or 2 to 3 interrupted open skin incisions are made with a scalpel along the SV course. Adventitial layers and surrounding connective tissue, including pericardial fat, are cleared meticulously with fine dissecting scissors. Side branches are divided after ligation or clipping to maintain a safe distance from the SV without narrowing the lumen. After reaching the needed length, the SV is divided, distally cannulated, and gently distended with physiologic solution by a syringe to test any remaining leaks or untreated side branches. The SV is stored at room temperature in saline, heparinized blood, or other storage solutions until later use. Systemic heparin is administered before cannulation for cardiopulmonary bypass in on-pump surgery or before the coronary anastomosis in off-pump surgery.

## NT SV Harvesting Techniques with or without Surrounding Pedicle Tissue

The NT SV Harvesting technique has been previously described.^6,8,14)^ Gundry et al. studied endothelial damage of SV grafts harvested without surrounding pedicle tissue and concluded that human SVs were preserved best by an NT harvesting technique in which manipulation was minimized and over-distention was avoided.^6)^ Dries et al. also studied optimal conditions of SV harvested without surrounding pedicle tissue and demonstrated that SVs receiving an NT dissection technique and not receiving luminal distention solution revealed significantly better endothelial preservation.^14)^ Souza suggested an NT SV dissection in which a cushion of surrounding tissues was left around the vein to prevent spasm and the deleterious effects of the contraction.^8)^

NT SV harvest is initiated after systemic heparinization during harvesting of the left ITA. Patients are given an initial dose of heparin (1.5 mg/kg) and periodic supplemental doses to maintain an activated clotting time of ≥300 seconds. Completely open or 2 to 3 interrupted open skin incisions are made with a scalpel along the SV course previously marked by surgical pens to avoid unnecessary and large dissections of the leg. Side branches are divided after clipping. Titanium clips are applied at least 1 mm from branch origins to prevent inadvertent endothelial injury. During NT SV harvesting, manipulation and tension of the SV are minimized, intraluminal dilatation or flushing using a syringe is strictly avoided, and perfusion of the SV is maintained. NT SV harvest can be performed either with or without the surrounding pedicle tissue. In the NT SV harvest without pedicle tissue, the SV is gently separated from the bed using scissors, leaving a small amount of perivascular adipose tissue in place. Manipulation with micro-forceps is limited to the perivascular tissue and never includes the full thickness of the vein wall. In the NT SV harvest with pedicle tissue, the SV pedicle is harvested along with an approximately 3- to 5-mm wide margin of adjacent adipose tissues on both sides of the SV and thin layers of adherent connective tissues posteriorly. NT SV harvest with pedicle tissue can be performed using either an electrocautery, Harmonic Scalpel (Ethicon Endo-Surgery Inc., Cincinnati, OH, USA), or LigaSure (Medtronic Inc., Minneapolis, MN, USA) device. During the NT SV harvest using an electrocautery, the cautery setting is kept on low throughout the dissection to avoid thermal injury to the SV pedicle. The NT SV harvest using either the Harmonic Scalpel or the LigaSure devices has the advantages of better hemostasis and minimal thermal injury over the NT SV harvest using an electrocautery. The Harmonic Scalpel device uses ultrasonic vibration technology, and the LigaSure device is an electrothermal bipolar-activated device. Both devices provide effective hemostasis and minimal lateral thermal spread. Immediately after the harvest and with no pharmacologic treatment, the reversed NT SV without or with pedicle tissue is anastomosed to the ascending aorta to construct a free SV graft or to the posterior aspect of the ITA to construct a Y-composite graft.^15,16)^

## Advantages of NT SV Harvesting

The NT technique protects the SV from direct handling that causes injury and spasm, avoids high-pressure distention, provides better preserved luminal endothelium and endothelial nitric oxide (NO) synthase, and maintains NO-producing capacity.^8,17,18)^ Preservation of the surrounding cushion of fat maintains the vasa vasorum, decreases transmural ischemic damage, and further preserves endothelial function.^18,19)^ Surrounding fat tissue is also an important source of various vasodilators and adipokines, including NO, leptin, and adiponectin.^20)^ In addition, the surrounding tissue acts as an external biological stent, protecting the SV wall against deleterious effects of arterial pressures and luminal distension, and maintains pulsatility of the cushioned graft, thereby reducing the neointimal and medial thickening and preventing the SV kinking.^21,22)^ Intravascular ultrasound studies showed a significantly slower progression of atherosclerosis in the NT SV compared with the conventionally harvested SV at 18-month and 8.5-year follow-up,^23)^ and insignificant differences in the proportion of intima–media area and the ratio of intima–media thickness in the NT SV compared with those in the left ITA at 1 year.^24)^ Postmortem biopsies at 8 to 18 years postoperatively revealed a less pronounced atherosclerotic process and adequate arterialization in the NT SV compared with the conventionally harvested SV.^25,26)^

## Patency Results of CABG Using NT SV as an Aortocoronary Graft

Souza et al., who introduced the NT SV harvested with surrounding pedicle tissue, performed a prospective randomized study to compare the patency rates of aortocoronary NT SV grafts with the patency rates of conventionally harvested SV aortocoronary grafts in 156 patients.^8,27)^ The patency rate of NT SV with surrounding pedicle tissue was higher than the patency rate of conventionally harvested SV at 18-month follow-up (95.4% vs 88.9%).^27)^ Longitudinal angiographic follow-up of the randomized study, which was performed in 74 patients of the 156 enrolled patients, showed that the NT SV patency rate was higher than the conventionally harvested SV patency rate at 8.5 years (90% vs 76%, *P* = 0.01), and was comparable to the ITA patency rate (90%).^28)^ Further angiographic follow-up of the randomized study, which included 54 patients of the 156 patients, showed that the patency rate of NT SV with surrounding pedicle tissue was significantly higher than that of the conventionally harvested SV (83% vs 64%, *P* = 0.03), and was comparable to that of the left ITA (88%) at 16 years postoperatively.^25)^ Another follow-up study of 2 randomized trials, which included 199 of 349 survived CABG patients using NT SV without surrounding pedicle tissue, showed a low occlusion rate of SV at 7 years (12.8%; 32 of 250 grafts).^29)^ In a network meta-analysis that included 14 randomized controlled trials, the NT SV was associated with lower graft occlusion compared with the conventionally harvested SV (incidence rate ratio, 0.55; 95% confidence interval [CI], 0.39–0.78).^30)^ A recent randomized, controlled, multicenter trial, which included 2655 patients, showed significantly lower occlusion rates in the NT SV with pedicle tissue than in the conventionally harvested SV at 12 months postoperatively (3.7% vs 6.5%; odds ratio, 0.56; 95% CI, 0.41– 0.76; *P* <0.001).^31)^

## Construction of the NT SV Y-Composite Graft

Immediately after the harvest, the reversed NT SV is anastomosed to the back side of the in-situ ITA without any pharmacologic treatment to construct a composite graft.^32)^ Most of the Y anastomoses are performed at the level of the pulmonary artery. After gripping the proximal ITA using an atraumatic bulldog clamp, an arteriotomy is made, and the SV is anastomosed to the ITA using an 8-0 polypropylene continuous suture. After construction of the SV composite graft and revascularization of the left anterior descending coronary artery using the in-situ left ITA, we destroyed the SV valve by inserting a 2-mm round-edge vessel dilator (much smaller than the diameter of the dilated SV) into the SV to prevent blood flow stagnation in a condition of flow competition. Destruction of the valve may cause endothelial injury in the SV graft; however, we believe the gain with valve destruction is greater than the losses without valve destruction.

## Patency Results of CABG Using the NT SV as a Composite Graft

Previous studies describing the use of SV composite grafts based on the left ITA demonstrated conflicting results. The study by Gaudino et al., in which the SV harvest method was not mentioned, included 25 patients who received an SV composite graft based on the left ITA. They recommended against the use of SV composite grafts because the SV composite grafts may steal flow from the left ITA and lead to suboptimal ITA perfect patency results (72% perfect patency at a mean of 2.5 years), although the SVs of the composite grafts were patent in all patients except one (96% patency).^33)^ The intraoperative flow dynamics study by Lobo Filho et al., in which NT SV was harvested without pedicle, demonstrated that the presence of SV composite grafts based on the left ITA did not alter the physiological blood flow dynamics in the distal segment of the left ITA.^34)^ Another hemodynamic study by Glineaur et al., in which the SV harvest method was not mentioned, showed that the hemodynamics of the right ITA composite grafts versus SV composite grafts based on the left ITA were similar in terms of pressure gradients at baseline and hyperemia, and fractional flow reserve at 6 months postoperatively.^35)^ A previous long-term observational study, in which a propensity score–matched analysis was used to match 103 patients who received SV composite grafts to 103 patients who received arterial composite grafts, showed no differences in overall or composite graft patency rates between the SV and arterial composite graft groups during the 10 years after surgery.^36)^ The authors harvested the SV using a minimal manipulation technique, which is an NT technique without surrounding pedicle tissue.^36)^ The 10-year angiographic results of the prospective randomized SAVE RITA trial, in which the SV was harvested using an NT technique without surrounding pedicle tissue, showed that the occlusion rate of the NT SV Y-composite graft was comparable with that of the right ITA composite graft at 10 years postoperatively (6.9%[12/173] vs 3.4% [5/146] *P* = 0.21).^12)^ Another observational study demonstrated that NT SV with pedicle tissue further improved the early and 1-year patency of SV composite grafts based on the ITA compared with those of NT SV without pedicle tissue, which might result from improved NT SV patency by maintaining pulsatility of the cushioned graft.^22)^

## Leg Wound Complication after NT SV Harvest

There have been concerns about increased leg wound complications after NT SV harvest, caused by the disruption of more tissues, including venous and lymphatic channels. Leg wound complication rates after NT SV harvest have been reported to be 6% to 23%, which are higher than the complication rates after conventional SV harvest.^27,31,37,38)^ Our strategies to minimize leg wound complications after NT SV harvest include: 1) assessment of iliofemoral vascular status by preoperative computed tomography angiography and avoiding SV harvest from the ipsilateral leg with severe peripheral arterial occlusive disease; 2) performing an ankle brachial index (ABI) test to avoid harvesting the ipsilateral SV if there is an ABI ratio of ≤0.7 in any lower limb; 3) using Doppler ultrasonography of the SV course for mapping an accurate SV location after anesthetic induction, thereby avoiding excessive soft tissue injury and the creation of tissue flap; 4) inserting a Jackson–Pratt drain into the SV harvesting site after transection of the SV; and 5) closing the leg wounds in layers using interrupted absorbable sutures to the subcutaneous tissue and additional staples or zip surgical closure method (ZipLine Medical Inc., Campbell, CA, USA) for the skin. The drain is removed when the amount of drained fluid decreases to <10 mL daily.^16)^ Using the above strategies, leg wound complications developed in 2 of 153 patients (1.3%) who received an NT SV graft from 2021 to 2023. The 2 patients suffered from partial wound disruption and recovered after secondary intention healing.^39)^

## Conclusions

Recent studies showed that the patency rates of NT SV grafts, without or with surrounding pedicle tissue, were excellent and comparable to those of the arterial grafts. Long-term clinical results of CABG using NT SV grafts are also excellent. Properly harvested and treated SV may perform well in the right environment.

## Disclosure statement

The authors declare that they have no conflicts of interest.
